# Perioperative Complications in Patients Undergoing Simultaneous Bilateral Lumboscopic Nephrectomy

**DOI:** 10.7759/cureus.93883

**Published:** 2025-10-05

**Authors:** Efrain Maldonado Alcaraz, Salvador Sanjuan Jimenez, Ashley Zulema Velazquez Jaimes

**Affiliations:** 1 Urology, Hospital de Especialidades "Dr. Bernardo Sepulveda" Centro Médico Nacional Siglo XXI, Mexico City, MEX

**Keywords:** kidney transplantation, laparoscopy, nephrectomy, postoperative complications, renal dialysis

## Abstract

Background and aim: Bilateral nephrectomy is necessary to optimize the condition of patients undergoing renal transplantation. Our simultaneous lumboscopic technique is scarcely documented but may offer benefits for patients with end-stage renal disease in the transplant protocol. This study aimed to identify major postoperative complications associated with this approach. This study aims to determine the frequency and characteristics of postoperative complications of grade ≥3, according to the Clavien-Dindo classification, in patients undergoing simultaneous bilateral lumboscopic nephrectomy.

Methodology: A retrospective cohort study was conducted on patients undergoing simultaneous bilateral lumboscopic nephrectomy with left nephroureterectomy and endoscopic bladder cuff excision, between January 2015 and February 2024. Clinical and surgical variables, major complications, and transfusion requirements were analyzed.

Results: Seventeen patients were included, with a mean age of 42.2 years and a female predominance (11, 64.7%). All were on renal replacement therapy. The most frequent indication was recurrent urinary tract infections associated with vesicoureteral reflux (11, 64.7%). The mean operative time was 228.2 minutes, with an average blood loss of 141.7 mL. According to the Clavien-Dindo classification, two grade III, two grade II, and three grade I complications were reported. Postoperative transfusions were required in 6 (35.3%) patients. No vascular or visceral complications occurred. Six patients successfully underwent renal transplantation. Conclusions: Simultaneous bilateral lumboscopic nephrectomy, including nephroureterectomy with bladder cuff excision, is a safe and feasible technique in renal transplant candidates, with an acceptable rate of major complications and preservation of the previous dialysis modality.

## Introduction

Optimizing renal transplant recipients by eliminating risk factors that may compromise graft survival is a key goal in pre-transplant preparation. Bilateral nephrectomy is indicated in certain candidates to help achieve this objective.

The procedure is commonly recommended in patients on transplant protocols who present with recurrent urinary tract infections, vesicoureteral reflux (VUR), autosomal dominant polycystic kidney disease, or treatment-resistant hypertension. In these cases, nephrectomy aims to eliminate chronic sources of infection and reduce immunologic or hemodynamic risks to the graft [1,2].

Several studies have shown that performing both nephrectomies in a single surgical session reduces the number of interventions, avoids prolonged anephric states, and may expedite the transplant process [3,4]. The retroperitoneal laparoscopic approach has demonstrated advantages in terms of recovery time, blood loss, and hospital stay compared to open surgery [5,6].

At our institution, the technique includes a left nephroureterectomy with endoscopic resection of the bladder cuff to avoid retention of an infected ureteral stump, particularly in cases of VUR. The right ureter is intentionally left intact to preserve the right iliac fossa for the future graft, minimizing surgical trauma and bleeding during transplantation [7,8].

Recent reports have described average operative times between 190 and 373 minutes, moderate blood loss, short hospital stays, and low rates of major complications with simultaneous bilateral nephrectomy [9,10]. However, there remains a need to better characterize postoperative complications-particularly those of greater clinical impact. This study focuses on complications graded ≥ III according to the Clavien-Dindo classification [11].

The main postoperative complications previously reported within three to seven months after nephrectomy include respiratory infections, postoperative bleeding, surgical site infections, vascular access thrombosis or deep vein thrombosis, and urinary fistulas such as uretero-cutaneous fistula or stump leakage [12].

The decision to proceed with surgical treatment and the choice of surgical technique are of critical importance, as these patients carry a high burden of comorbidities, making them particularly susceptible to postoperative complications.

This highlights the importance of considering a simultaneous bilateral approach in order to reduce intraoperative and postoperative complications, as well as to lower hospitalization and operating room costs.

To our knowledge, this is the only published case series describing simultaneous bilateral lumboscopic nephrectomy that includes a left nephroureterectomy with endoscopic bladder cuff excision in renal transplant candidates with VUR.

## Materials and methods

Study design

We conducted a retrospective cohort study at the Department of Urology, Hospital de Especialidades “Dr. Bernardo Sepúlveda Gutiérrez”, Centro Médico Nacional Siglo XXI (Mexico City, Mexico). The study aimed to evaluate perioperative complications in patients undergoing simultaneous bilateral lumboscopic nephrectomy as part of the pre-transplant protocol. The study period ranged from January 2015 to February 2024. This study was approved by the institutional review board and complied with ethical standards for retrospective observational research.

Patient recruitment

We included all patients with end-stage renal disease (ESRD) who underwent simultaneous bilateral lumboscopic nephrectomy at our institution during the study period. The surgical indication was based on recurrent urinary tract infections, vesicoureteral reflux, polycystic kidney disease, or severe hypertension as part of the transplant preparation protocol.

All patients aged 18 years or older who underwent simultaneous bilateral lumboscopic nephrectomy between January 2015 and February 2024 were considered eligible for inclusion. Patients were included if the procedure was performed in a single surgical session and if their clinical records and perioperative data were complete. Patients were excluded if they had incomplete documentation, underwent staged (non-simultaneous) nephrectomies, or required conversion to open surgery during the procedure.

Data collection

Patient data were extracted manually from physical and electronic medical records and entered into a standardized database. Demographic variables (age, sex, weight, height), clinical history (comorbidities, dialysis modality, urologic history), surgical variables (operative time, blood loss, complications), and postoperative outcomes (hospital stay, transfusion requirement, complications by Clavien-Dindo classification) were collected and analyzed. Preoperative laboratory data were also obtained, including hemoglobin, platelet count, and serum creatinine.

Sample size justification

As this is a retrospective descriptive study, a formal sample size calculation was not performed. The final sample included all consecutive eligible patients who underwent the procedure during the study period, maximizing the power of the available data. This approach has been applied in prior similar studies evaluating rare surgical techniques and outcomes.

Surgical technique

All procedures were performed under general anesthesia by the same surgical team. The surgery began with the patient in the lithotomy position. A 26 Fr resectoscope was used to perform endoscopic excision of the left ureteral orifice with the bladder cuff. The patient was then repositioned to a left flank oblique decubitus position to perform the left nephroureterectomy via retroperitoneal access. A balloon-dilated optical trocar was inserted through the Petit triangle, and two additional trocars were placed: one 5 mm trocar triangulated toward the iliac crest and one 12 mm trocar at the tip of the 11th rib. Cephalocaudal dissection of the kidney and ureter was completed, with ureteral detachment at the previously resected bladder cuff. Vascular control was achieved with Hem-o-lok clips, and a Penrose drain was placed.

Subsequently, the patient was repositioned to a right flank oblique decubitus position, and a right simple nephrectomy was performed using the same retroperitoneal technique and trocar configuration.

Statistical analysis

Descriptive statistical analysis was performed using IBM SPSS Statistics version 24 for Mac (IBM Corp., Armonk, NY). Quantitative variables were expressed as mean ± standard deviation (SD) or median and range, depending on data distribution. Categorical variables were reported as frequencies and percentages. No inferential statistics were applied, given the observational, descriptive nature of the study.

## Results

A total of 17 patients underwent simultaneous bilateral lumboscopic nephrectomy, for a total of 34 renal units removed. The mean age was 42.2 ± 11.6 years, with a predominance of female patients (11, 64.7%). All patients were on renal replacement therapy at the time of surgery. The most common surgical indication for nephrectomy was vesicoureteral reflux associated with recurrent urinary tract infections (64.7%). It is important to note that this percentage (7, 41.2%) reflects the primary indication for surgery, while the presence of urinary tract infection (UTI) as an isolated comorbidity - regardless of surgical indication - is reported separately in Table 1. Preoperative demographic characteristics are shown in Table 1.

All patients resumed their previous dialysis modality postoperatively without complications. No intraoperative vascular, bowel, or adjacent organ injuries were reported. Two patients experienced incidental peritoneal opening during dissection, which was managed without clinical consequences. The mean preoperative hemoglobin level was 10.0 g/dL, while the postoperative mean was 8.8 g/dL. These values are reported descriptively without statistical comparison, in accordance with the observational design of the study. At the time of data collection, six patients (35.2%) had already undergone successful renal transplantation. Surgical metrics and complications: mean operative time, 228.2 ± 41.5 minutes; mean anesthetic time, 292 ± 45.8 minutes; mean estimated blood loss, 141.7 ± 106.6 mL; mean hospital stay, 4.0 ± 2.2 days; median Foley catheter duration, 2.0 days (range 0-10); median drainage output, 15 mL (range 0-61). Postoperative transfusion was required in six patients (35.3%), while two patients (11.8%) received intraoperative transfusions. In all cases, the transfusion was limited to a single unit of packed red blood cells. Clavien-Dindo classification: grade I, 3 (17.6%); grade II, 2 (11.8%); grade III, 2 (11.8%). No grade IV or V complications were observed. Postoperative characteristics are shown in Table 2.

**Table 1:** Preoperative demographic characteristics. This table presents the baseline characteristics of the 17 patients included in the study. Demographic data include age, sex, body weight, and height. Clinical variables include the presence of comorbidities such as hypertension and diabetes, history of peritonitis, urinary tract infections, and prior surgical procedures. Preoperative laboratory values and dialysis modality (peritoneal vs. hemodialysis) are also shown. Surgical indications for nephrectomy are listed, including vesicoureteral reflux and polycystic kidney disease. Values are reported as mean ± standard deviation, median (range), or frequency (%), as appropriate. ASA, American Society of Anesthesiologists

Variable	Result
Age (years), mean (SD)	42.2 (11.6)
Gender	
Male, *n* (%)	6 (35.3)
Female, *n* (%)	11 (64.7)
Weight (kg), median (range)	58 (53-77)
Height (cm), mean (SD)	156.6 (79.7)
Comorbidities, *n* (%)	
Diabetes mellitus type 2	2 (11.8)
Systemic arterial hypertension	15 (88.2)
History of peritonitis, *n* (%)	12 (70.6)
History of urinary tract infection, *n* (%)	7 (41.2)
History of previous surgery, *n* (%)	16 (94.1)
History of previous urological surgeries, *n* (%)	1 (5.9)
Type of renal replacement therapy, *n* (%)	
Peritoneal dialysis	7 (41.2)
Hemodialysis	10 (58.8)
Indications for nephrectomy, *n* (%)	
Vesicoureteral reflux (VUR)	11 (64.7)
Polycystic kidney disease	2 (11.8)
Urinary malformations	2 (11.8)
VUR, renal cysts, nephrolithiasis	1 (5.9)
VUR, malformations, nephrolithiasis	1 (5.9)
Incomplete bladder emptying, *n* (%)	4 (23.5)
Positive urine culture, *n* (%)	3 (17.6)
Anuria, *n* (%)	11 (64.7)
Preoperative hemoglobin (mg/dL), mean (SD)	10.0 (1.8)
Preoperative platelets (10*3/uL), mean (SD)	213.3 (71.0)
Preoperative creatinine (mg/dL), mean (SD)	13.0 (3.7)
ASA, *n* (%)	
ASA II	10 (58.8)
ASA III	2 (0-4)

**Table 2:** Postoperative characteristics. This table summarizes key intraoperative and postoperative outcomes following simultaneous bilateral lumboscopic nephrectomy. Variables include total operative time, anesthetic time, estimated blood loss, hospital stay duration, Foley catheter time, and drainage output. Laboratory values such as postoperative hemoglobin and need for blood transfusion are included. Incidental peritoneal opening and postoperative complications are classified according to the Clavien-Dindo grading system. Results are presented as mean ± standard deviation, median (range), or frequency (%).

Variable	Result
Length of stay (days), mean (SD)	4.0 (2.2)
Time of transurethral catheter (days), median (range)	2.0 (0.0-10.0)
Surgical time (minutes), mean (SD)	228.2 (41.5)
Anesthetic time (minutes), mean (SD)	292 (45.8)
Surgical blood loss (mL), mean (SD)	141.7 (106.6)
Surgical drainage output (mL), median (range)	15 (0.0 - 61)
Postoperative hemoglobin, mean (SD)	8.8 (1.5)
Transurgical transfusion, *n* (%)	2 (11.8)
Post-surgical transfusion, *n* (%)	6 (35.3)
Incidental peritoneal opening, *n* (%)	2 (11.8)
Clavien-Dindo complications, *n* (%)	
Clavien-Dindo I	3 (17.6)
Clavien-Dindo II	2 (11.8)
Clavien-Dindo III	2 (11.8)

## Discussion

To our knowledge, this is the largest published case series of simultaneous bilateral lumboscopic nephrectomies and the first to describe a right simple nephrectomy combined with a left nephroureterectomy and endoscopic bladder cuff excision in renal transplant candidates with VUR as the most frequent indication.

Our findings align with previous literature in terms of operative time, blood loss, and complication rates. Jarzemski et al. reported an operative time range of 150 to 240 minutes, with a mean of 139 minutes, and an average blood loss of 142 mL using a retroperitoneal laparoscopic approach in a small cohort of seven patients [10]. In our series, the mean operative time was longer (228.2 minutes), likely due to the inclusion of bladder cuff excision and the need for intraoperative repositioning. The reported blood loss was comparable (141.7 mL), although a significant proportion of patients required transfusion. This finding is consistent with the known bleeding risk in patients with end-stage renal disease and highlights the importance of careful perioperative management, rather than serving as a direct indicator of surgical safety.

Regarding complication rates, our study showed 17.6% grade I, 11.8% grade II, and 11.8% grade III complications according to Clavien-Dindo. These findings are consistent with the ranges reported in previous studies on bilateral nephrectomy in dialysis patients. For example, Grodstein et al. evaluated the safety of simultaneous bilateral nephrectomy and renal transplantation and reported a surgical site complication rate of 25% when nephrectomy and transplant were performed in the same session [13]. This supports our preference for a staged approach, allowing for hematologic recovery (i.e., restoration of hemoglobin levels and stabilization of hematologic parameters) and minimizing graft-related risks.

In terms of transfusion requirements, 35.3% of patients in our cohort required postoperative transfusions, typically limited to a single unit of packed red blood cells. No cases required multiple units or massive transfusion. This rate is consistent with the elevated risk of bleeding in patients with chronic kidney disease due to platelet dysfunction and anemia [4,13]. Despite this, no major vascular or visceral injuries were reported, and all patients resumed their prior dialysis modality postoperatively-an important outcome in institutions with high rates of peritoneal dialysis.

Other studies, such as those by Kramer et al., have reported success with combining bilateral nephrectomy and transplantation in patients with polycystic kidney disease, but they also noted increased intraoperative complexity and longer recovery times [9]. In contrast, our approach aims to separate nephrectomy from transplantation to simplify graft placement, particularly by preserving the right iliac fossa.

Additionally, the inclusion of bladder cuff excision in patients with VUR may offer an advantage by eliminating residual ureteral segments that could become sources of infection or inflammation. Wu et al. and Attalla et al. have emphasized the importance of complete ureteral excision in oncologic contexts, and while our setting is non-malignant, the principle of eliminating infected or dysfunctional tissue remains valid [7,8].

Finally, although our sample size is modest, it represents a focused and uniform cohort. However, the absence of a control group and the retrospective nature of the study limit its internal validity and should be considered when interpreting the results. The use of a standardized surgical technique and uniform postoperative care across all patients strengthens the reliability of the outcomes presented.

Limitations

This study has some limitations. First, the retrospective and single-center design may limit the generalizability of our findings. Second, the relatively small sample size restricts the ability to perform inferential statistical analysis or identify predictors of complications. Third, the absence of a control group limits comparison with alternative approaches such as staged nephrectomy or combined nephrectomy-transplant procedures. The exact volume of blood transfused per patient was not consistently documented in the medical records, which limits the ability to quantify total transfusion burden. Finally, the potential for selection bias cannot be excluded, as all cases were selected based on surgical indication within a specialized transplant program.

## Conclusions

Simultaneous bilateral lumboscopic nephrectomy, including left nephroureterectomy with endoscopic bladder cuff excision, is a feasible and safe procedure in renal transplant candidates. It is associated with an acceptable rate of major complications and allows patients to maintain their previous dialysis modality. Our findings suggest that this technique is feasible and safe in our institutional setting and may warrant further investigation in other transplant centers, particularly for patients with recurrent urinary infections and vesicoureteral reflux.

## References

[1] Darby CR, Cranston D, Raine AE, Morris PJ (1991). Bilateral nephrectomy before transplantation: indications, surgical approach, morbidity and mortality. Br J Surg.

[2] Caione P, Ciofetta G, Collura G, Morano S, Capozza N (2004). Renal damage in vesico-ureteric reflux. BJU Int.

[3] Martin AD, Mekeel KL, Castle EP (2012). Laparoscopic bilateral native nephrectomies with simultaneous kidney transplantation. BJU Int.

[4] Hofer RE, Kor TM, Prieto M, Findlay JY (2021). The perioperative management of simultaneous bilateral nephrectomy with renal transplantation: a case series. Can J Anaesth.

[5] Guillonneau B, Ballanger P, Lugagne PM, Valla JS, Vallancien G (1996). Laparoscopic versus lumboscopic nephrectomy. Eur Urol.

[6] Rassweiler J, Frede T, Henkel TO, Stock C, Alken P (1998). Nephrectomy: a comparative study between the transperitoneal and retroperitoneal laparoscopic versus the open approach. Eur Urol.

[7] Wu Z, Li M, Wang J (2021). Pure retroperitoneoscopic extravesical standardized seeable (PRESS) excision of distal ureter and bladder cuff in radical nephroureterectomy: step-by-step technique. Minerva Urol Nephrol.

[8] Attalla K, Patnaik S, Vellos T, Mehrazin R (2019). Management of distal ureter and bladder cuff at the time of nephroureterectomy: surgical techniques and predictors of outcome. Future Oncol.

[9] Kramer A, Sausville J, Haririan A, Bartlett S, Cooper M, Phelan M (2009). Simultaneous bilateral native nephrectomy and living donor renal transplantation are successful for polycystic kidney disease: the University of Maryland experience. J Urol.

[10] Jarzemski P, Listopadzki S, Słupski P, Jarzemski M, Brzoszczyk B (2020). Simultaneous bilateral native nephrectomy by retroperitoneal approach. Int Braz J Urol.

[11] Dindo D, Demartines N, Clavien PA (2004). Classification of surgical complications: a new proposal with evaluation in a cohort of 6336 patients and results of a survey. Ann Surg.

[12] Ou CH, Yang WH (2011). Bilateral hand-assisted retroperitoneoscopic nephroureterectomy (HARN) in the spread-eagle position for dialysis patients-low midline HARN in a completely supine position. Urology.

[13] Grodstein EI, Baggett N, Wayne S (2017). An evaluation of the safety and efficacy of simultaneous bilateral nephrectomy and renal transplantation for polycystic kidney disease: a 20‑year experience. Transplantation.

